# P-274. Rates of Extended-Spectrum Beta-Lactamase-Producing Enterobacterales across Tennessee, 2019 – 2022

**DOI:** 10.1093/ofid/ofae631.478

**Published:** 2025-01-29

**Authors:** Ashley Gambrell, Srilakshmi Velrajan, Dipen Patel, Melphine Harriott, Daniel Muleta

**Affiliations:** Tennessee Department of Health, Nashville, Tennessee; Tennessee Department of Health, Nashville, Tennessee; HAI/AR, Tennessee Department of Health, Nashville, Tennessee; TN Department of Health, Nashville, Tennessee; Tennessee Department of Health, Nashville TN, Antioch, Tennessee

## Abstract

**Background:**

Extended-spectrum beta-­­lactamase (ESBL)-producing Enterobacterales were responsible for an estimated 197,400 infections and 9,100 deaths amongst hospitalized patients in 2017 in United States. Surveillance of ESBL-producing Enterobacterales, as a part of Multi-site Gram Negative Surveillance Initiative (MuGSI) project was conducted in four Tennessee counties (Maury, Marshall, Lewis, and Wayne). Little is known specifically about the rates of ESBL-Enterobacterales geographically over these areas. We calculated and compared rates at the county and census tract level for the TN MuGSI catchment area, for 2019 – 2022.

Prevalence Rates of ESBL-Enterobacterales Cases by Tennessee County and Census Tract

**Figure 1.**
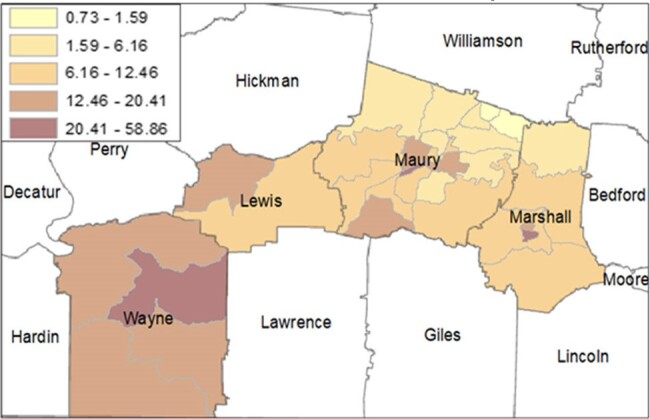
Rates of Cases of ESBL-producing Enterobacterales by County and Census Tract, 2019 – 2022. Rates are calculated per 1,000 residents for each census tract and grouped by natural breaks classification method. County and census tract boundaries are shown in grey outline outline.

**Methods:**

Data from MuGSI Case Detection System for 2019 – 2022 were geocoded to the county and census tract level using ArcMap. Population counts, including demographics, were taken from the U.S. Census Bureau’s 2020 Census. Analysis was completed in SAS 9.4.

**Results:**

At the county-level, the average prevalence rate was 16.76 cases per 1,000 residents. Wayne county had the highest prevalence at 29.69 cases per 1,000 residents, while Maury county had the lowest, at 9.39 cases per 1,000 residents. Prevalence rates amongst the census tracts varied widely, with a mean of 13.04 cases per 1,000 residents, and ranging from 0.73 cases up to 58.86 cases (Figure 1). Wayne County’s 4 census tracts consistently showed the highest rates, ranging from 13.48 to 58.86 cases per 1,000 residents, while Maury County’s 21 tracts had the lowest rates, ranging from 0.73 to 31.98 cases per 1,000 residents. From 2019 to 2022, most counties had stable prevalence rates, however Wayne County showed an increase from 3.60, to 9.60 cases per 1,000 residents by 2022.

**Conclusion:**

Rates of ESBL-producing Enterobacterales varied substantially, even amongst a relatively small catchment area within the state. Highest rates of this pathogen were found in census tracts from Wayne and Lewis County, with Wayne County also showing a large increase of cases from 2019 to 2022. Understanding of what drives rates disproportionately in these areas is crucial to prevention and mitigation, not only for ESBL-producing Enterobacterales, but for many other gram-negative multidrug resistant organisms. More research is needed to highlight the drivers of these disparate rates.

**Disclosures:**

**All Authors**: No reported disclosures

